# Disease severity accounts for minimal variance of quality of life in people with dementia and their carers: analyses of cross-sectional data from the MODEM study

**DOI:** 10.1186/s12877-020-01629-1

**Published:** 2020-07-06

**Authors:** Nicolas Farina, Derek King, Clare Burgon, Sharne Berwald, Elizabeth Bustard, Yvonne Feeney, Ruth Habibi, Adelina Comas-Herrera, Martin Knapp, Sube Banerjee, On behalf of the MODEM group, Bayo Adelaja, Mauricio Avendano, Sally-Marie Bamford, Sube Banerjee, Sharne Berwald, Ann Bowling, Clare Burgon, Elizabeth Bustard, Ruth Habibi, Adelina Comas-Herrera, Margaret Dangoor, Josie Dixon, Nicolas Farina, Yvonne Feeney, Sally Greengross, Emily Grundy, Bo Hu, Carol Jagger, Kate Jopling, Martin Knapp, Derek King, Andrew Kingston, Daniel Lombard, Klara Lorenz, David McDaid, A-La Park, Jitka Pikhartova, Sanna Read, Amritpal Rehill, Raphael Wittenberg

**Affiliations:** 1 Centre for Dementia Studies, Brighton and Sussex Medical School, Brighton, UK; 2 London School of Economics and Political Science, London, UK; 3 Faculty of Medicine & Health Sciences, University of Nottingham, Nottingham, UK; 4 Faculty of Health, University of Plymouth, Plymouth, UK

**Keywords:** Quality of life, Dementia, Alzheimer’s disease, Wellbeing, Health, Cognitive impairment, Caregiver

## Abstract

**Background:**

Due to the progressive nature of dementia, it is important to understand links between disease severity and health-related outcomes. The aim of this study is to explore the relationship between disease severity and the quality of life (QoL) of people with dementia and their family carers using a number of disease-specific and generic measures.

**Methods:**

In the MODEM cohort study, three-hundred and seven people with clinically diagnosed dementia and their carers were recruited on a quota basis to provide equal numbers of people with mild (standardised Mini-Mental State Examination (sMMSE), *n* = 110), moderate (sMMSE 10–19, *n* = 100), and severe (sMMSE 0–9, *n* = 97) cognitive impairment. A series of multiple regression models were created to understand the associations between dementia severity and the QoL of people with dementia and the QoL of their carers. QoL was measured using self- (DEMQOL, EQ-5D, CASP-19) and proxy-reports (DEMQOL-Proxy, EQ-5D) of disease-specific and generic QoL of the person with dementia. Carer generic QoL was measured by self-report (EQ-5D, SF-12).

**Results:**

Disease severity, as measured by the sMMSE, was not significantly associated with the QoL of the person with dementia or the carer (*p* > 0.05), even after controlling for potential confounding variables for self-reported instruments. Proxy measures (rated by the carer) differed systematically in that there were small, but statistically significant proportions of the variance of QoL was explained by severity of cognitive impairment in multiple adjusted models. We also found little in the way of statistically significant relationships between the QoL of people with dementia and that of their carers except between DEMQOL-Proxy scores and the carer EQ-5D scores and carer SF-12 mental sub-scores.

**Conclusions:**

The data generated supports the somewhat counterintuitive argument that severity of cognitive impairment (and therefore severity of dementia) is not associated with lower QoL for the person with dementia when self-report measures are used. However, in absolute terms, as judged by the variance in the multivariate models, it is clear that the contribution of dementia severity to the QoL of people with dementia is minimal whatever the measurement used, be it self- or proxy-rated, or disease-specific or generic.

## Background

Dementia is a progressive neurodegenerative disorder that has a profound impact on individuals, their family, and society. As dementia progresses, functional ability and cognitive function decline with a consequent increase in the amount of care required [1]. This can lead to substantial personal and societal costs [2]. During the course of the illness other complications can arise unpredictably including neuropsychiatric symptoms such as agitation, aggression, depression and anxiety [3, 4].

In the UK it is estimated that, in terms of severity, just over half of people with dementia (55.4%) have mild dementia, 32.1% have moderate dementia, and 12.5% have severe dementia [5]. Understanding the impact of disease severity on dementia -related outcomes is important to enable the development and delivery of treatment and care that allows people to live well with dementia and for families and healthcare providers to respond to changes as the disease progresses. The goal of such care is to maintain and enhance the quality of life (QoL) of the person with dementia and this is therefore a key outcome to understand and measure. Understanding the impact of disease severity on carer-related outcomes is equally as important as it ensures that the carer’s own QoL does not suffer as a result of the inevitable deterioration of the person for whom they provide care.

Quality of life is a multidimensional construct that incorporates an “individual’s perception of their position in life in the context of culture and value systems in which they live and in relation to their goals, expectations, standards, and concerns” (pg 1405) [6]. For people with dementia, demographic factors do not appear to have a strong effect on QoL [7–9]; instead neuropsychiatric symptoms (e.g. depression) appear to account for greater proportions of variance [9–11]. Intuitively, we may assume that as the severity of dementia increases, exemplified by cognitive and functional decline, so does the QoL of the person with dementia decline. However, reviews of the literature have found mixed evidence about the relationship between QoL and severity of cognitive impairment in people with dementia [8, 12], with some authors finding that increased cognitive impairment predicts lower QoL [13], whilst others have found no such relationship [14]. It has previously been suggested that such differences in findings could be explained in part by the use of self-report versus proxy-report measures of QoL [8].

Similarly, it has also been assumed that increasing disease severity would negatively impact on family carer QoL, due to changes in personal freedom of the carer, practical demands, and its impact on the interpersonal relationship [15]. There is less literature in the field of carer QoL, but again to date most studies have (apparently paradoxically) found no association between carer QoL and the severity of cognitive impairment of the person with dementia [16]. Instead, factors such as behavioural problems, carer burden and depression appear to be more consistently associated with carer QoL [16–18] However, there may be an indirect effect between disease severity and carer QoL through increasing carer burden and depression [19].

The MODEM programme was designed to model how changes in the treatment and care of people with dementia, and support for carers, can result in better outcomes [20]. One element of this was the collection of new, high-quality, one-year cohort data from people with dementia and their carers. Participants were purposively recruited to capture samples balanced in numbers across disease severity, based on cognitive impairment. Unlike many previous studies, we sought to capture a range of measures of QoL because one purpose of the cohort study was to collect data that would allow cross-walk between measures used in epidemiological surveys and in clinical evaluations.

In this paper we investigate the baseline characteristics of the MODEM cohort, with a focus on understanding the relationship between disease severity and the QoL of the person with dementia and their carer. It was hypothesised that dementia severity would not be associated with the QoL of the person with dementia or their carer after controlling for confounding variables.

## Methods

### Design

Cross-sectional baseline data from a cohort study.

### Participants

Participants had a medical diagnosis of dementia made by a specialist mental health service. We aimed to recruit 100 people with mild (scoring 20+ on the standardised Mini-Mental State Examination), 100 with moderate (score 10–19), and 100 with severe cognitive impairment (score 0–9). These severity categories are in line with recommendations for categorising moderate and severe dementia using the sMMSE [21]. To be eligible the person with dementia needed to have an identifiable family (or friend) carer or other informant (e.g. a formal/professional carer). There were no exclusion criteria based on comorbidities, age, or type of dementia. The carer required to self-identify themselves as a carer for the person with dementia, there were no other inclusion or exclusion criteria. Participants were recruited from memory services in Sussex, UK, or self-referral from a national electronic database (Join Dementia Research; https://www.joindementiaresearch.nihr.ac.uk/), community groups, and care homes in the South East of England.

### Procedure

People with dementia and their carers were provided with information about the research and invited to participate in the study. A pair of researchers then visited the participants in their home (or another location convenient for the participant). The capacity of the person with dementia was formally assessed by a trained researcher. A form was also completed, which guided the researcher to make judgements on whether the participant was a) able to understand the purpose of the study, b) able to retain the information long enough to make a decision, c) able to weigh up the information in order to make a decision, and d) able to communicate their decision. If the person with dementia did not have capacity to consent, a personal consultee (family member/friend) was identified to advise on whether the person with dementia should take part. For those with capacity, informed consent was obtained. The two researchers then completed a series of measures with the person with dementia and the carer in parallel. Additional context and an overview of the methods have been reported elsewhere [20]. Ethical Approval was obtained by the Social Care Research Ethics Committee (15/IEC08/0005).

### Measures

The following measures were completed. They can be divided into three response formats: 1) person with dementia completed measures about themselves, 2) carer completed about the person with dementia, and 3) carer completed about themselves. Broadly, QoL can be measured using either generic or disease-specific strategies [22]. The generic strategy uses instruments applicable across different diseases and treatments, at the cost of perhaps failing to address disease-specific elements that may be crucial to QoL in a given condition, so being less sensitive in detecting changes in outcome [22, 23]. Disease-specific approaches can complement these generic outcomes by providing insight into the complex nature of the condition, which might change as the disease progresses [14, 24, 25].

### Self-report measures of QoL (person with dementia)

1. DEMQOL [26] – 28 item interviewer-administered questionnaire answered by the individual with dementia, a dementia-specific health-related QoL measure. DEMQOL data were collected for mild and moderate dementia as per its instructions for use. The measure was developed for use in people with dementia, has good internal consistency (α = 0.87) and moderate convergent validity [24, 26]. The total score of the DEMQOL ranges from 28 to 112, with higher scores representing better QoL.
2. EQ-5D-3L [27] – a 5 item, self-report questionnaire on generic health related QoL. The EQ-5D-3L is able to distinguish between healthy and a range of disease groups [28]. The self-report measure has been shown to be feasible for use within people with mild-to-moderate dementia [29]. A summary index score was calculated using country-specific value sets, which generally range from 0 (death) to 1 (perfect health). Data were not collected for those with severe dementia.
3. CASP-19 [30] – a 19 item, self-report measure of QoL comprising four domains (control, autonomy, self-realisation and pleasure). In people with dementia, the CASP-19 display good overall internal consistency (α = 0.86) and correlates with an established measure of QoL (*r* = 0.71) [31]. Individual items are summed together and range from 0 to 57, in which higher scores represent a “total satisfaction of control, autonomy, self-realization and pleasure domains”. Data were not collected for those with severe dementia.

### Proxy-report measures of QoL (person with dementia)

1. DEMQOL-Proxy [26] – 31 item interviewer-administered questionnaire answered by the carer on the individual with dementia, a dementia-specific health-related QoL measure. The measure demonstrates high internal consistency in mild-moderate severity dementia (α = 0.87) and in severe dementia (α =0.92) [26]. Individual items are summed to create a total score (Range: 31–124), with higher scores representing better QoL.
2. EQ-5D-3L [27] – a 5 item, proxy-report questionnaire on generic health related quality of life. Inter-rater agreement between the self-and proxy report version of the EQ-5D-3L are typically quite low [29]. See above for further details about the EQ-5D-3L measure.

### Self-report measures of QoL (Carer)

1. EQ-5D-3L [27]– a 5 item, self-report questionnaire on generic health related quality of life. See above for further details about the EQ-5D-3L measure.
2. Short Form Health Survey (SF-12) [32] – a 12 item questionnaire to measure generic health related quality of life. Two summary measures are calculated, the physical health and mental health domain, both of which have shown to have high internal consistency across populations (e.g. α = 0.85 and 0.83, respectively in an elderly population) [33]. SF-12 summary scores each range from 0 to 100, with 100 indicate the highest level of health.

### Other measures

1. Standardized Mini-Mental State Examination (sMMSE) [34] - a brief, global measure of cognitive function. The MMSE generally displays good internal consistency (e.g. α = 0.70) [35] and maps onto dedicated dementia staging questionnaires such as the Clinical Dementia Rating scale [36, 37]. The sMMSE is a standardized version of the MMSE, in terms of how it is administered and scored. Correct items are summed to create a total score ranging from 0 to 30, with lower scores representing greater cognitive impairment and severity.
2. Zarit Carer Burden Inventory (ZCBI) [38] – a 22 item scale to measure carer burden. The questionnaire has a high internal consistency in carers of people with dementia (α = 0.90) [39]. Individual items are summed (Range: 0–88), with higher scores reflect greater carer burden.
3. Neuropsychiatric Inventory (NPI) [40, 41] – an instrument to record presence of 10 behavioural and psychological symptoms in dementia. The total score has a high level of internal consistency (α = 0.88), and displays an good level of concurrent validity with an existing validated measure of behavioural and psychological symptoms in dementia [41]. The total score is calculated by multiplying frequency by severity for each domain, and summing them together. Higher scores represent more behavioural and psychological symptoms (Range: 0–120).

### Analysis

The mean and standard deviation (SD) were reported for continuous data, frequencies and percentages were reported for categorical data. All descriptive data were split by severity (sMMSE total score: 20–30 = mild; 10–19 = moderate; 0–9 = severe). Preliminary between group analyses were completed using a Pearson’s Chi Square test for categorical data, and an ANOVA or Student’s t-test for continuous data (three-group and two-group comparisons, respectively).

QoL was our primary domain of interest, captured through a battery of self-reports (DEMQOL, EQ-5D, CASP-19) and proxy-reports (DEMQOL-Proxy, EQ-5D) of disease-specific and generic QoL for the person with dementia, and generic self-reported QoL (EQ-5D, SF-12) for carers. We completed a Pearson’s correlation analysis between outcomes to explore how they were associated with one another. We then identified whether the associations remained significant following correction for multiple comparisons [42].

A series of multiple regression models were created, in which QoL outcomes were the dependent variables. In each model, factors were entered into three stages. At Stage 1, a simple model was created with sMMSE as the only independent variable. At Stage 2, control variables were entered into the model to control for their effects, without the presence of sMMSE. At Stage 3, the sMMSE scores were entered in the model following the control variables. This allowed us to identify the amount of explained variance in severity after accounting for the control variables.

For analyses of the QoL of people with dementia, variables entered in Stage 2 were age of the person with dementia, their gender, neuropsychiatric symptoms (NPI total score), carer QoL (EQ-5D) and residential setting. For carer QoL outcomes, the following variables were entered in Stage 2; carer age, carer gender, relationship with person with dementia, residential setting of the person with dementia, neuropsychiatric symptoms of the person with dementia, carer burden (ZCBI), and the QoL of person with dementia (DEMQOL-Proxy). Keeping the variables consistent between models within outcome themes allowed us to compare the differential role of disease severity.

Variables entered in the model were selected on the basis on existing evidence, and consensus within the experienced research team. In selecting the measures, we were conscious that we wanted to control for key confounding effects, rather than build a “best fit” model. As such, we avoided the use of step-wise regression, which is susceptible to inflating type I error [43], or including all potentially confounding variables with the risk of over-fitting the model [44].

We visually inspected regression outputs (e.g. P-P plot) for evidence of normality of residuals of the regression and homoscedasticity. Multicollinearity was checked between independent variables prior to building the models: a Variance Inflation Factor (VIF) threshold of 10 was used to indicate probable multicollinearity [45]. There were no instances of the VIF exceeding 3.

Missing values within standardised questionnaires were handled in accordance with measure guidance, when available. Listwise deletion was adopted across analyses in the case of missing values. Statistical significance was defined as a *p* value < 0.05. All data were analysed in SPSS V.25.

## Results

Three hundred and seven participant dyads were consented into the study. Of those with dementia, 110 were classified as having mild dementia, 100 moderate, and 97 severe. On average participants had a sMMSE of 14.9 (SD = 9.08). 155 (50.5%) of the people with dementia were female, the average age of the sample was 80.9 years old (SD = 8.42) and most were diagnosed with Alzheimer’s disease (*n* = 176, 60.7%). Carers tended to be female (*n* = 202, 65.8%), spousal carers (*n* = 198, 64.7%), and their average age was 69.2 years (SD = 11.45). For detail of demographic and other key variables, please see Tables 1 and 2.

**Table 1:** Person with dementia, descriptive data by severity of cognitive impairment

	Mild	Moderate	Severe	Sig. Test
**Age, mean (SD)**	80.2 (7.2)	82.4 (8.8)	80.0 (9.2)	F = 2.50, *p* = 0.08
**Gender: male, N (%)**	62 (56.4%)	55 (55.6%)	34 (35.1%)	χ^2^ = 11.62, *p* = 0.003
**Residential status: care home (v own household), N (%)**	6 (5.5%)	17 (17.0%)	40 (41.2%)	χ^2^ = 41.59, *p* < 0.0001
**Ethnicity: White British, N (%)**	105 (96.3%)	88 (89.8%)	88 (90.7%)	χ^2^ = 13.03, *p* = 0.37
**Diagnosis:**				χ^2^ = 4.28, *p* = 0.83
**AD, N (%)**	63 (63.0%)	55 (57.9%)	58 (61.1%)	
**VaD, N (%)**	13 (13.0%)	12 (12.6%)	14 (14.7%)	
**LBD, N (%)**	2 (2.0%)	1 (1.1%)	3 (3.2%)	
**FTD, N (%)**	4 (4.0%)	4 (4.2%)	1 (1.1%)	
**Other, N (%)**	18 (18.0%)	23 (24.2%)	19 (20.0%)	
**sMMSE (↑ better cognition), mean (SD)**	24.6 (0.2)	15.1 (2.9)	3.6 (3.2)	F = 1360.36, *p* < 0.0001
QOL SELF-REPORT				
**DEMQOL (↑ better QoL), mean (SD)**	90.5 (13.18)	92.8 (12.6)	–	t = −1.23, *p* = 0.22
**CASP total (↑ better QoL), mean (SD)**	40.9 (9.5)	40.1 (8.0)	–	t = 0.58 *p* = 0.61
**EQ-5D Index (↑ better QoL), mean (SD)**	0.8 (0.2)	0.8 (0.2)	–	t = −0.08, *p* = 0.94
QOL PROXY-REPORT				
**DEMQOL-Proxy (↑ better QoL), mean (SD)**	93.5 (13.7)	94.3 (14.4)	98.1 (13.0)	F = 3.18, *p* = 0.04
**EQ-5D-proxy Index (↑ better QoL), mean (SD)**	0.7 (0.3)	0.5 (0.3)	0.4 (0.3)	F = 26.76, *p* < 0.0001
COMORBIDITIES				
**NPI total (↑ more neuropsychiatric symptoms), mean (SD)**	12.7 (11.0)	20.1 (15.5)	24.1 (16.3)	F = 15.51, *p* < 0.0001

*AD* Alzheimer’s Disease, *BADL* Bristol Activities of Daily Living, *CCI* Charlson Comorbidity Index, *FTD* Frontotemporal Dementia, *LBD* Lewy Body Dementia, *NPI* Neuropsychiatric Inventory, *QoL* Quality of Life, *sMMSE* standardised Mini-Mental State Examination, *OARS* Older Adult Social Resource Scale, *VaD* Vascular Dementia

F = F statistic derived from one-way Analysis of Variance, t = t value derived from t-test, χ^2^ = Chi-square value derived from a Pearson’s Chi-square test

**Table 2:** Carer, descriptive data by severity of cognitive impairment

	Mild	Moderate	Severe	Sig. Test
**Age, mean (SD)**	71.5 (12.0)	68.7 (11.4)	67.3 (10.6)	F = 3.64, *p* = 0.03
**Gender: male, N (%)**	32 (29.4%)	26 (26.5%)	44 (45.4%)	χ^2^ = 9.09, *p* = 0.01
**Ethnicity: White British, N (%)**	106 (98.1%)	90 (91.8%)	89 (94.1%)	χ^2^ = 11.38, *p* = 0.33
**Live with person with dementia: Yes, N (%)**	87 (79.8%)	66 (67.3%)	50 (51.5%)	χ^2^ = 18.51, *p* < 0.001
**Relationship to Person with dementia: Spousal, N (%)**	84 (76.4%)	60 (60.6%)	54 (55.7%)	χ^2^ = 13.43, *P* = 0.009
**Zarit Carer Burden Inventory (↑ greater burden), mean (SD)**	28.6 (13.9)	32.0 (16.2)	29.5 (15.7)	F = 1.40, *p* = 0.25
**EQ-5D self (↑ better QoL), mean (SD)**	0.8 (0.2)	0.8 (0.3)	0.9 (0.2)	F = 2.00, *p* = 0.14
**SF-12: Physical (↑ better health/QoL), mean (SD)**	48.2 (11.5)	46.9 (12.4)	49.4 (10.8)	F = 1.11, *p* = 0.33
**SF-12: Mental (↑ better health/QoL), mean (SD)**	47.9 (10.2)	45.3 (10.0)	46.8 (11.0)	F = 1.49, *p* = 0.23

*GHQ* General Health Questionnaire

F = F statistic derived from one-way Analysis of Variance, t = t value derived from t-test, χ^2^ = Chi-square value derived from a Pearson’s Chi-square test

Table 3 presents correlations between the QoL measures used for people with dementia and carers. For self-report of QoL by the person with dementia (DEMQOL, CASP-19, EQ-5D) there were statistically significant correlations between all measures (*p* < 0.001) which remained statistically significant after correcting for multiple comparisons (Holm-Bonferroni Sequential Correction, *p* < 0.05). For proxy-reports, the disease-specific measure DEMQOL-Proxy was statistically significantly correlated with all self-report measures of the QoL of the person with dementia (DEMQOL, EQ-5D, CASP-19) at the *p* < 0.001 level, with this remaining after correction for multiple comparison. The generic proxy measure (EQ-5D) was correlated statistically significantly univariately with all the self- and proxy-reported instruments (DEMQOL, EQ-5D (self), CASP-19 (self), DEMQOL-Proxy), but when correction for multiple comparison was applied the association with CASP-19 (self) and DEMQOL-Proxy did not reach statistical significance.

**Table 3:** Correlations between person with dementia and carer QoL measures (Pearson’s r and *p*-values)

		Person with dementia				Carer (all self-reports)		
		EQ-5D (self)	CASP-19 (self)	DEMQOL-Proxy	EQ-5D (proxy)	Carer EQ-5D	SF-12 Physical	SF-12 Mental
**Person with dementia**	**DEMQOL (self)**	**0.56 (*p*** **< 0.001)**	**0.72 (*p*** **< 0.001)**	**0.39 (*p*** **< 0.001)**	**0.20 (*p*** **= 0.003)**	0.11 (*p* = 0.13)	0.10 (*p* = 0.19)	0.09 (*p* = 0.23)
	**EQ-5D (self)**		**0.58 (*p*** **< 0.001)**	**0.33 (*p*** **< 0.001)**	**0.36 (*p*** **< 0.001)**	0.12 (*p* = 0.09)	0.14 (*p* = 0.06)	0.18 (*p* = 0.02)
	**CASP-19 (self)**			**0.33 (*p*** **< 0.001)**	0.21 (*p* = 0.01)	0.09 (*p* = 0.29)	0.11 (*p* = 0.19)	0.06 (*p* = 0.49)
	**DEMQOL-Proxy**				0.16 (*p* = 0.005)	**0.20 (*p*** **= 0.001)**	0.13 (*p* = 0.03)	**0.31 (*p*** **= < 0.001)**
	**EQ-5D (proxy)**					0.02 (*p* = 0.77)	−0.01 (*p* = 0.92)	0.07 (*p* = 0.28)
**Carer**	**EQ-5D**						**0.71 (*p*** **< 0.001)**	**0.25 (*p*** **< 0.001)**
	**SF-12 Physical**							−0.05 (*p* = 0.41)

Statistics in bold highlight remained significant after correcting for multiple comparisons (Holm-Bonferroni Sequential Correction, *p* < 0.05)

For carer QoL, the carer EQ-5D score and SF-12 mental sub-score were statistically significantly (after multiple comparison correction) associated with the DEMQOL-Proxy score for the person with dementia. The only other correlation that persisted after correction was between the carer EQ-5D and SF-12 physical and mental sub-scores.

### Person with dementia quality of life (self-report)

Dementia severity as measured by the sMMSE did not significantly contribute to explanation of variation in DEMQOL scores in the uncontrolled model (*β* = − 0.12, *p* = 0.09). After controlling for other variables, sMMSE still did not significantly contribute to DEMQOL scores, accounting for only 2% of variance (*β =* − 0.15*,* ΔR^2^ = 0.02, *p* = 0.06). No covariates were significantly associated with DEMQOL scores. See Table 4.

**Table 4:** Multiple regression models with person with dementia QoL outcomes as dependent variables. The model fit is reported for each model, and the contribution of disease severity (sMMSE) reported (ΔR^2^ and *p*-value). Individual variables beta coefficients (including 95% CIs) are also reported

		Coefficients					Model change		Model		
Model	Independent Variables	β	B	B Lower 95% CI	B Upper 95% CI	p	ΔR^2^	p	Adjusted R^**2**^	F	p
**DEMQOL** (*n* = 177)							0.02	0.06	0.04	2.19	0.05
	Gender: Male	−0.11	−2.97	−6.92	0.98	0.14					
	Residential setting: Care home	−0.13	−5.26	−11.73	1.22	0.11					
	Age	0.15	0.24	−0.01	0.49	0.06					
	NPI	− 0.05	−0.05	−0.19	0.09	0.49					
	Carer EQ-5D	0.07	3.51	−3.81	10.84	0.35					
	sMMSE	−0.15	−0.37	−0.76	0.01	0.06					
**EQ-5D (self)** (*n* = 185)							0.001	0.63	0.00	0.99	0.43
	Gender: Male	−0.03	− 0.01	− 0.08	0.06	0.75					
	Residential setting: Care home	−0.01	0.00	−0.12	0.11	0.95					
	Age	0.04	0.00	0.00	0.01	0.60					
	NPI	−0.13	0.00	0.00	0.00	0.09					
	Carer EQ-5D	0.10	0.09	−0.05	0.22	0.21					
	sMMSE	−0.04	0.00	−0.01	0.00	0.63					
**CASP-19 (self)** (*n* = 155)							< 0.001	0.89	0.03	1.63	0.14
	Gender: Male	−0.10	−1.81	−4.91	1.29	0.25					
	Residential setting: Care home	−0.12	−3.98	−9.73	1.77	0.17					
	Age	0.21	0.25	0.05	0.45	0.02					
	NPI	−0.10	−0.07	−0.20	0.06	0.27					
	Carer EQ-5D	0.02	0.61	−5.26	6.47	0.84					
	sMMSE	0.01	0.02	−0.29	0.34	0.89					
**DEMQOL-Proxy** (*n* = 284)							0.06	< 0.001	0.22	14.14	< 0.001
	Gender: Male	0.07	2.01	−0.96	4.98	0.18					
	Residential setting: Care home	−0.01	−0.39	−4.52	3.75	0.85					
	Age	0.00	0.01	−0.17	0.19	0.95					
	NPI	−0.43	−0.39	−0.49	− 0.29	0.00					
	Carer EQ-5D	0.14	8.11	2.09	14.13	0.01					
	sMMSE	−0.29	−0.44	−0.62	− 0.26	0.00					
**EQ-5D (proxy)** (*n* = 276)							0.04	< 0.001	0.25	16.36	< 0.001
	Gender: Male	0.13	0.08	0.01	0.15	0.02					
	Residential setting: Care home	−0.16	−0.13	−0.23	−0.03	0.01					
	Age	−0.13	− 0.01	−0.01	0.00	0.02					
	NPI	−0.26	−0.01	−0.01	0.00	0.00					
	Carer EQ-5D	0.08	0.10	−0.04	0.24	0.15					
	sMMSE	0.23	0.01	0.00	0.01	0.00					

Dementia severity was not significantly associated with self-report EQ-5D scores in the uncontrolled model (*β* = − 0.02, *p* = 0.83). Dementia severity did not significantly account for variance of the self-report EQ-5D after controlling for confounding variables (*β =* − 0.04, ΔR^2^ = 0.001, *p* = 0.63). No variables within the model were significantly associated with self-report EQ-5D scores. See Table 4.

Dementia severity was not significantly associated with CASP-19 scores in the uncontrolled model (*β* = 0.04, *p* = 0.64). For the CASP-19, dementia severity again did not significantly account for variance in the model after accounting for control variables (*β =* − 0.01, ΔR^2^ < 0.001, *p* = 0.89). Only age was significantly associated with CASP-19 scores in the model, with older age being associated with improved QoL (*β* = 0.21, *p* = 0.02). See Table 4.

### Person with dementia quality of life (proxy-report)

The uncontrolled model revealed that sMMSE scores were negatively associated with DEMQOL-Proxy scores (*β* = − 0.18, *p* = 0.001). Severity was associated with DEMQOL-Proxy scores, accounting for 6% of variance in the controlled model (*β* = − 0.29, ΔR^2^ = 0.06, *p* < 0.001). Worse cognitive function was associated with significantly higher DEMQOL-Proxy scores within the model, indicating that QoL by proxy assessment was higher in the groups of people with more severe dementia. There was a significant negative association between DEMQOL-Proxy scores and NPI scores (*β* = − 0.43, *p* < 0.001) with higher levels of neuropsychiatric symptoms associated with lower QoL for the person with dementia and carer self-reported QoL (*β* = 0.14, *p* = 0.01). See Table 4.

Severity was also statistically significantly associated with proxy-reported EQ-5D scores in both the uncontrolled (*β =* 0.42, *p* < 0.001) and controlled model (*β =* 0.23, ΔR^2^ = 0.04, *p* < 0.001). Participants with better cognitive function had significantly higher EQ-5D Proxy scores. See Table 4. Within the model, being older (*β* = − 0.13, *p* = 0.02), living in a care home (*β* = − 0.16, *p* = 0.01), and having neuropsychiatric symptoms (*β* = − 0.26, *p* < 0.001) were significantly associated with poorer QoL as measured by the proxy-report EQ-5D. Whilst being male was associated with better QoL scores (*β* = 0.13, *p* = 0.02). This suggests differential functioning of disease-specific and generic QoL measures.

### Carer quality of life

Severity of dementia was significantly associated with carer self-report EQ-5D, in the absence of control variables (*β* = − 0.13, *p* = 0.03). This association did not remain significant after controlling for confounding variables, explaining only 1% of the variance (*β =* − 0.09*,* ΔR^2^ = 0.01, *p* = 0.19). Within the model, carer burden was significantly negatively associated with EQ-5D (*β* = − 0.28, *p* < 0.001; i.e. the higher the carer burden the lower the QoL). The QoL of the person with dementia was also positively associated with EQ-5D in the model (*β* = 0.14, *p* = 0.03). No other variable was significantly associated with carer EQ-5D in the model. See Table 5.

**Table 5:** Multiple regression models with carer-related QoL measures as dependent variables. The model fit is reported for each model, and the contribution of disease severity (sMMSE) reported (ΔR^2^ and *p*-value). Individual variables beta coefficients (including 95% CIs) are also reported

		Coefficients					Model change		Model		
Model	Independent variables	β	B	B Lower 95% CI	B Upper 95% CI	p	ΔR^2^	p	Adjusted R^**2**^	F	p
**EQ-5D carer** (*n* = 213)							0.01	0.19	0.12	5.83	< 0.001
	Carer gender: Male	0.08	0.04	−0.02	0.10	0.19					
	Residential setting: Care home	−0.09	− 0.05	−0.13	0.03	0.19					
	Carer age	−0.13	0.00	−0.01	0.00	0.10					
	Relationship: Spouse	−0.04	−0.02	−0.10	0.07	0.66					
	NPI	0.08	0.00	0.00	0.00	0.24					
	ZCBI	−0.28	−0.01	−0.01	−0.00	0.00					
	DEMQOL-Proxy	0.14	0.00	0.00	0.01	0.03	`				
	sMMSE	−0.09	−0.00	−0.01	0.00	0.19					
**SF-12 Physical Health** (*n* = 273)							0.003	0.37	0.05	2.80	0.01
	Carer gender: Male	0.02	0.54	−2.47	3.54	0.72					
	Residential setting: Care home	−0.12	−3.44	−7.64	0.75	0.11					
	Carer age	−0.17	−0.18	− 0.35	−0.01	0.04					
	Relationship: Spouse	−0.03	−0.79	−5.09	3.52	0.72					
	NPI	0.03	0.02	−0.10	0.14	0.70					
	ZCBI	−0.16	−0.13	−0.23	− 0.02	0.02					
	DEMQOL-Proxy	0.09	0.08	−0.04	0.19	0.18					
	sMMSE	−0.0	−0.08	−0.27	0.10	0.37					
**SF-12 Mental Health** (*n* = 273)							< 0.001	0.97	0.43	26.56	< 0.001
	Carer gender: Male	0.07	1.57	−0.50	3.64	0.14					
	Residential setting: Care home	−0.09	−2.46	−5.35	0.43	0.10					
	Carer age	0.16	0.14	0.02	0.26	0.02					
	Relationship: Spouse	−0.05	−1.03	−3.99	1.94	0.50					
	NPI	−0.07	−0.05	−0.13	0.03	0.22					
	ZCBI	−0.57	−0.39	−0.46	− 0.32	0.00					
	DEMQOL-Proxy	0.13	0.10	0.02	0.18	0.02					
	sMMSE	0.00	0.00	−0.13	0.13	0.97					

For the SF-12, severity did not significantly account for variance of either physical (*β =* − 0.08, *p* = 0.19) or mental health domains (*β =* 0.01, *p* = 0.93) in the uncontrolled models. After controlling for confounding variables, both physical (*β* = − 0.06, *p* = 0.37) and mental health (*β* = 0.002, *p* = 0.97) each accounted for less than 1% of variance. For the mental health domain, carer age (*β* = 0.16, *p* = 0.02), carer burden (*β* = − 0.57, *p* < 0.001), and DEMQOL-Proxy (*β* = 0.13, *p* = 0.02) were all significantly associated with the outcome. Only carer age (*β* = − 0.17, *p* = 0.04) and carer burden (*β* = − 0.16, *p* = 0.02) were significantly associated with the SF-12 physical domain. See Table 5.

## Discussion

This study adds to the literature by taking multiple assessments of the QoL of people with dementia and their carers, and examining their association with severity of dementia (as measured by severity of cognitive impairment). Our analyses support the somewhat counterintuitive argument that severity of cognitive impairment (and therefore severity of dementia) is not associated with lower QoL for the person with dementia when self-report measures are used. The data suggest that, after controlling for key variables, the QoL of people with dementia may even improve as the condition deteriorates. However, this was only statistically significant in a single proxy-report measure (DEMQOL-Proxy) and approached significance (*p* = 0.06) in a single self-report measure (DEMQOL). Proxy measures (rated by the carer) differed systematically in that there were small but statistically significant proportions of the variance of QoL explained by severity of cognitive impairment in multiple adjusted models. Previous research has indeed found that proxy-reported outcomes tend to differ to self-report outcomes, with the proxies reporting more health and functional impairments [46, 47]. There are three main possible reasons for this. First, the greater range of severity of dementia that can be included using proxy measures (self-reports were only possible in the mild and moderate severity groups) means that particular harms that may accrue in the very latest stages of dementia may contribute to the findings. Second there is known error in making proxy assessments of another’s QoL, and it may be that such proxy assessments are over-influenced by the intuitive belief that increasing severity of dementia must have a negative impact on the person with dementia. Third, it is likely that lack of insight, or lack of engagement with their illness, could result in people with dementia under-reporting reduced QoL. Certainly, impaired insight provides less reliable ratings of QoL [48], and may even effect the reliability and validity of some self-report measures of QoL [49]. However, in absolute terms, as judged by the variance in the multivariate models, it is clear that the contribution of dementia severity to the QoL of people with dementia is minimal whatever the measurement used, be it self- or proxy-rated, or disease-specific or generic. Similarly, when examining the relationship between carer QoL and severity of dementia, in multivariate analyses there were no associations between greater dementia severity and lower carer QoL as measured by the EQ-5D or SF-12, with the variance largely driven by the severity of carer burden. We also found little in the way of statistically significant relationships between the QoL of people with dementia and that of their carers except between DEMQOL-Proxy scores and the carer EQ-5D scores and carer SF-12 mental sub-scores.

The finding that severity is not associated with dementia-specific measures of QoL supports previous evidence both cross-sectionally and longitudinally [50–52]. While there was no association with severity in self-report measures, proxy-report measures of QoL (DEMQOL-Proxy and proxy-report EQ-5D) did show a statistically significant association with severity. As noted above, proxy measures are of particular importance in studies of people with more severe dementia because of greater difficulties with self-report. For example, DEMQOL’s psychometrics are strong for those with mild and moderate dementia but not for those with severe dementia, so it cannot be used reliably in those with severe cognitive impairment [26]. As such, for people with severe dementia, we are unable to know the associations between the score of sMMSE with their self-reported QoL. Limitations in awareness of cognitive impairment, or lack of insight, are often cited as reasons why self-reported QoL does not decline as disease severity increases, with older adults who are aware of their dementia diagnosis rating their QoL lower than those that were unaware [53]. This is in line with previous literature that highlights the effect of awareness of disease and depressive symptoms on self-report QoL scores [54]. However, even with limitations in insight, these self-appraisals of QoL may well be valid. People with dementia may not be much concerned with gradual deterioration in cognitive and physical function to which they and their families have been able to adapt. This is in line with the findings of adaptation to other long-term conditions with subsequent high life quality, often termed ‘the disability paradox’ [55]. Discrepancies between DEMQOL-Proxy and proxy EQ-5D may be due to the nature of the measures, since EQ-5D focusses on functionality and ability, rather than emotional responses in DEMQOL-Proxy. In this way, the EQ-5D may act as a measure of function, which undoubtedly declines with increasing cognitive impairment, rather than QoL, which may not. The differences in what is actually measured may also explain the weak association between the two measures (*r* = 0.16). Such emphasis on functional ability when measuring QoL using generic measures and those not developed and validated for dementia has been criticised elsewhere [56].

Neuropsychiatric symptoms, as measured by the NPI, were significantly associated with scores across all proxy-report QoL instruments, but not in self-report QoL instruments. This is in line with previous findings which are either negative [8, 57], or account for only a small amount of variance [7]. A recent meta-analysis of the literature found that neuropsychiatric symptoms showed a moderate association with poorer QoL, irrespective of rating type [52]. However, as noted by Naglie and colleagues, certain QoL measures may be more sensitive than others [58].

For carer QoL, severity of cognitive impairment in the person with dementia was not significantly associated with scores, accounting for less than 2% of variance within the models. The only factor across carer QoL outcome measures to be consistently associated with carer QoL was the ZCBI which is a measure of subjective carer burden. This is in line with the literature that consistently shows a negative association between carer QoL and subjective reports of carer burden [16]. This indicates that the carer’s perceived burden of care may be a more important factor in determining QoL than severity of dementia alone, and therefore supporting carers is paramount to maintain good carer QoL.

Carer QoL was significantly associated with age (SF-12 physical and mental health domains). Interestingly age was positively associated with carer QoL in the mental health domain, but negatively associated with physical health domain. These same associations have been reported elsewhere [59–61], although it is still unclear whether this is due to differential item functioning (e.g. different interpretation of items and differences in response style). Carer QoL (EQ-5D and SF-12 mental health domain) was significantly associated with the QoL of the person with dementia, supporting previous findings [17, 62–64]. However, as previously highlighted, there may be an element of reverse causality, because carers may project their own QoL and health state into their assessment of the person with dementia [16].

A strength of this paper is the simultaneous use of a number of QoL tools. In MODEM, we used both generic QoL measures (EQ-5D and CASP) and disease-specific measures of QoL (DEMQOL, DEMQOL-Proxy). However, due to an absence of robustly developed disease-specific carer QoL outcomes specific to dementia [65] only generic measures of carer QoL were available. This supports a need for dementia-specific carer QoL outcome measures.

An important limitation of the study is that the people with dementia and the carers recruited were predominantly White British (92.4 and 94.1% respectively). While this reflects the general population of older adults in England and Wales [66], the relative lack of ethnic diversity limits the generalisability of our findings, particularly to other cultures and countries. Results reported here reflect a modest sample of people with dementia (and their carers) who were motivated to participate in this research project within South East England. The study does not include people with dementia who do not have a carer, and whilst this would have prevented proxy-report measurement, we are unable to comment whether dementia severity would differentially effect those that are living independently. We did not capture the response rate to the study, and therefore unable to directly quantify any non-response bias introduced. It is also important to recognise that these findings only describe a cross-sectional relationship between severity and QoL; the analyses need to be replicated longitudinally to investigate the effects of the progressive nature of dementia.

The models presented here should not be considered as definitive models of factors associated with QoL. There are potentially other variables that could have accounted for additional variance within each model (e.g. insight [67]). This is particularly the case for self-report QoL measures of people with dementia, where very little variance (< 5%) is accounted for. Finally, it should be noted that the use of sMMSE as the measure of severity could be considered a limitation. The sMMSE measures cognitive impairment, rather than dementia severity specifically, although obviously the two are closely linked, with the MMSE mapping onto measures of severity [36, 37]. Despite MMSE being widely adopted, it has received criticism, such as not being education-fair, and suffering from floor and ceiling effects [68–71].

## Conclusions

Our findings highlight the complex relationship between severity of dementia and QoL in people living with dementia and their carers. They highlight the effect of tool selection of outcomes. Proxy-report measures of dementia-specific QoL are more strongly associated with dementia severity. It may well be that relatively slow deterioration in dementia allows for the processes of adaptation seen in other disabilities to allow people with severe dementia to live as a good quality of life as those with less severe dementia. We see the same picture in relation to carer QoL: increased severity of impairment of the person with dementia does not necessarily mean poorer carer QoL, and instead factors such as carer burden play a much greater central role.

## Data Availability

Data will be made available upon reasonable request to the corresponding author.

## Abbreviations

NPI: Neuropsychiatric Inventory
QoL: Quality of Life
SF-12: Short Form Survey
sMMSE: Standardised Mini-Mental State Examination
VIF: Variance Inflation Factor
ZCBI: Zarit Carer Burden Inventory
